# Effect of Kangaroo-mother care combined with infant touch on premature infants

**DOI:** 10.12669/pjms.41.5.10187

**Published:** 2025-05

**Authors:** Qiuying Du, Hongbin Zhu, Man Guo, Huichan Yang, Yanfei Ma

**Affiliations:** 1 Qiuying Du Department of Paediatrics, Maternity & Child Care Center of Qinhuangdao, Qinhuangdao 066000, Hebei, China; 2 Hongbin Zhu Department of Paediatrics, Maternity & Child Care Center of Qinhuangdao, Qinhuangdao 066000, Hebei, China; 3 Man Guo Department of Paediatrics, Maternity & Child Care Center of Qinhuangdao, Qinhuangdao 066000, Hebei, China; 4 Huichan Yang Department of Paediatrics, Maternity & Child Care Center of Qinhuangdao, Qinhuangdao 066000, Hebei, China; 5 Yanfei Ma Department of Paediatrics, Maternity & Child Care Center of Qinhuangdao, Qinhuangdao 066000, Hebei, China

**Keywords:** Development, Infant touch, Nutritional status, Premature infants, Kangaroo-mother care, Sleep quality

## Abstract

**Objective:**

To explore the effect of Kangaroo-mother care combined with infant touch on premature infants.

**Methods:**

This was a retrospective study. Ninety two premature infants in Maternity & Child Care Center of Qinhuangdao from July 2022 to July 2023 were included. The enrolled patients were randomly divided into a control group (routine nursing intervention) and an observation group (Kangaroo-mother care combined with infant touch) using a random number table method (n=46 each group). Further comparison was made on physical development, nutritional status, and sleep quality between groups.

**Results:**

There was no statistically significant difference in body height and weight between the two groups of infants at birth(*P*>0.05). The body height and weight of the two groups of infants both increased at 3 days, 7 days and 30 days after birth, which were higher in the observation group than those in the control group(*P*<0.05). Furthermore, the levels of zinc, iron, and prealbumin in the observation group were significantly higher than those in the control group at 30 days and 90 days after birth, respectively(*P*<0.05). Moreover, the good sleep quality rate of infants in the observation group was significantly higher than that in the control group (63.04% vs. 36.96%), while the poor sleep quality rate of infants in the former group was obviously lower than that in the latter group (8.70% vs. 26.09%), with statistically significant differences(*P*<0.05).

**Conclusion:**

Kangaroo-mother care combined with infant touch has a significant effect on premature infants, which can promote their physical development, improve their nutritional status and sleep quality.

## INTRODUCTION

Premature infants should be nursed more professionally due to early birth and incomplete development, requiring special attention paid to and support for physical-psychological health.^1^-^3^ Conventional nursing interventions cannot meet the needs of premature infants. Kangaroo-mother care and infant touch both emphasize on promoting healthy growth of infants. Specifically, the former nursing type, also known as skin-to-skin contact, mimics the maternal environment of the fetus to enhance the baby’s sense of security, which can promote sleep and reduce anxiety.^4^-^6^ Infant touch can promote infant growth and development by gently stimulating the infant’s skin for physical stimulation. This stimulus can stimulate the infant’s sensory systems such as touch, vision, and hearing, thereby promoting the development and improvement of their nervous system.^7^,^8^ Kangaroo-mother care combined with infant touch can better meet the needs of premature infants.^9^ However, there are currently few studies on the effect of Kangaroo-mother care combined with infant touch on premature infants. Therefore, this study was performed by including 92 premature infants in Maternity & Child Care Center of Qinhuangdao from July 2022 to July 2023 to explore the impact of Kangaroo-mother care combined with infant touch on premature infants.

## METHODS

This was a retrospective study. Ninety two premature infants in Maternity & Child Care Center of Qinhuangdao from July 2022 to July 2023 were randomly divided into a control group (routine nursing intervention) and an observation group (Kangaroo-mother care combined with infant touch) using a random number table method, with 46 cases in each group.

### Ethics approval:

The study was approved by the Institutional Ethics Committee of Maternity & Child Care Center of Qinhuangdao (No. QHDFY-2023041910; date: April 19, 2023), and written informed consents were provided by the legal guardians of enrolled children.

### Inclusion criteria:

- Infants with gestational age of 28~36 weeks;
- Infants with Apgar scores of 7-9 points;
- Infants whose family members had provided written informed consent.

### Exclusion criteria:

- Infants with congenital malformations
- Puerpera with mental disorders, cognitive impairments, etc. who were unable to communicate normally;
- Puerpera who were unable to feed normally due to infectious diseases and other factors.

### Control group:

Infants in this group were given routine nursing intervention, including

1. ***Living environment:*** Premature infants should live in a quiet, clean, and moderately warm environment with fresh air but no convective winds;
2. ***Feeding:*** With sufficient nutritional supply required, premature infants should be breastfed as much as possible and provided with premature infant formula if necessary. Moreover, attention should be paid to observe the presence of spitting up after feeding;
3. ***Skin care:*** Premature infants have delicate skin, and hence it was important to keep the skin clean and dry. The diaper should be soft and changed frequently to prevent diaper rash;
4. ***Warming:*** Premature infants have poor thermoregulation ability, and therefore it is necessary to strengthen the management of warming. In general, the room temperature should be maintained between 24-28°C, and the humidity should be between 55%-65%; and
5. ***Health protection:*** Premature infants have weak immunity, and it was important to pay attention to hygiene protection to avoid cross infection.

### Observation group:

Infants in this group were provided with Kangaroo-mother care combined with infant touch, including: Kangaroo-mother care: Skin-to-skin contact between the mother and infant by keeping the infant in the mother’s arms can maintain the infant’s body temperature, promote neurodevelopment, alleviate pain, etc. Specifically, 1-2 hours after infant feeding, the indoor temperature can be controlled between 22-24°C, with soft and comfortable sofas or lounge chairs, baby hats and other items prepared at the same time. After removing infant clothes, changing diapers, and putting on a baby hat, the infant was placed in a prepared kangaroo-style nursing bag to have skin contact with the mother; and infant touch: On the basis of Kangaroo-mother care, infant touch can be performed gently to promote the infant’s neurodevelopment, reduce pain, increase the infant’s sense of security and self-confidence, etc. The touch involved the head, chest, abdomen, limbs, and back, with each area being touched for approximately 5-10 minutes. It was important to wash hands and cut nails short before touching, and keep the infant comfortable and relaxed when touching.

### Outcome measures:

### Physical development:

The body height and weight of newborns were recorded at birth, three days, seven days, and 30 days after birth.

### Nutritional status:

The levels of zinc, iron, and prealbumin in newborns were recorded at 30 days and 90 days after birth.

### Sleep quality:

Good sleep quality: Newborns had a continuous sleep duration of three hours, can fall asleep quickly, had no waking up during sleep, no significant crying during sleep, and a continuous sleep duration of 2-3 hours; normal sleep quality: Newborns had occasional crying and waking up during sleep, and a continuous sleep duration of only one hour; and poor sleep quality: Newborns frequently cried and woke up during sleep. Further recording and comparison were made on night-time awakening duration, night-time awakening frequency, night-time sleeping duration, and day-time sleeping duration between groups.

### Statistical analysis:

By using SPSS22.0 software for statistical analysis, measurement data that conformed to normal distribution were expressed as (*χ̅±S*) and compared by t-test; while categorical variables were presented by cases and percentages, and compared using χ^2^ test. A *P*-value of <0.05 indicated the presence of statistically significant difference.

## RESULTS

In the observation group, there were 23 males and 23 females, with an average gestational age of (31.39±1.00) weeks (28~34 weeks), including 25 cases of natural delivery and 21 cases of cesarean section. While in the observation group, there were 22 males and 24 females, with an average gestational age of (31.54±1.17) weeks (28~35 weeks), including 27 cases of natural delivery and 19 cases of cesarean section. As shown in Table-I, there was no statistically significant difference in the comparison of general data between the two groups (*P*>0.05).

**Table-I:** Comparison of general data between groups [*n*(%), *χ̅±S*].

Groups	n	Gender		Gestational age (weeks)	Mode of delivery	
		Male	Female		Natural delivery	Cesarean section
Observational group	46	23 (50.00)	23 (50.00)	31.39±1.00	25 (54.35)	21 (45.65)
Control group	46	22 (47.83)	24 (52.17)	31.54±1.17	27 (58.70)	19 (41.30)
*χ^2^* /*t*	-	0.043		0.671	0.177	
*P* value	-	0.835		0.504	0.674	

There was no statistically significant difference in body height and weight between the two groups of infants at birth (*P*>0.05). Table-II, The body height and weight of the two groups of infants both increased at 3 days, 7 days and 30 days after birth, which were higher in the observation group than those in the control group (*P*<0.05). In Table-III, the levels of zinc, iron, and prealbumin in the observation group were significantly higher than those in the control group at 30 days and 90 days after birth, respectively (*P*<0.05).

**Table-II:** Comparison of physical development between groups (*χ̅±S*).

Groups	n	Time point	Body height (cm)	Body weight (g)
Observation group	46	At birth	40.29±0.51	1900.40±100.18
		3 days after birth	40.76±0.59*^#^	1970.50±108.21*^#^
		7 days after birth	43.09±0.71*^#^	2584.80±320.18*^#^
		30 days after birth	45.56±0.79*^#^	4252.50±310.21*^#^
Control group	46	At birth	40.20±0.48	1893.49±100.51
		3 days after birth	40.40±0.51*	1943.46±120.49*
		7 days after birth	41.60±0.58*	2312.09±240.21*
		30 days after birth	43.20±0.41*	3742.06±350.19*

*Note:*

* compared at the same time point within the same group, ^*^P<0.05; #compared with the control group,

# *P*<0.05.

**Table-III:** Comparison of nutritional status between groups (*χ̅±S*).

Groups	n	Zinc (umol/L)		Iron (umol/L)		Prealbumin (mg/L)	
		30 days after birth	90 days after birth	30 days after birth	90 days after birth	30 days after birth	90 days after birth
Observation group	46	12.11±1.60	15.33±2.08*	3.29±0.71	4.40±0.68*	11.66±0.41	16.08±2.39*
Control group	46	10.03±1.46	13.56±1.50*	2.76±0.59	3.10±0.71*	10.70±1.78	14.46±2.48*
*t*		6.512	4.682	3.891	8.982	3.565	3.190
*P*		<0.001	<0.001	<0.001	<0.001	0.001	0.002

*Note:*

* compared at 30d after birth within the same group, *P<0.05.

The good sleep quality rate of infants in the observation group was significantly higher than that in the control group (63.04% vs. 36.96%), while the poor sleep quality rate of infants in the former group was obviously lower than that in the latter group (8.70% vs. 26.09%), with statistically significant differences (*P*<0.05). In addition, the observation group had shorter night-time awakening duration and lower frequency of night-time awakenings than those in the control group, and longer night-time and day-time sleep duration than those in the latter group. Table-IV, Table-V.

**Table-IV:** Comparison of sleep quality between groups.

Groups	n	Good	Normal	Poor
Observation group	46	29 (63.04)	13 (28.26)	4 (8.70)
Control group	46	17 (36.96)	17 (36.96)	12 (26.09)
*χ^2^*	-	6.261	0.791	4.842
*P*	-	0.012	0.374	0.028

**Table-V:** Comparison of sleep quality between groups (*χ̅±S*).

Groups	n	Night-time awakening duration (hours)	Night-time awakening frequency (times)	Night-time sleeping duration (hours)	Day-time sleeping duration (hours)
Observation group	46	2.87±0.62	2.70±0.47	11.90±0.56	6.27±0.93
Control group	46	3.48±0.43	3.11±0.53	9.88±1.28	5.35±0.56
*χ^2^*	-	-5.483	-3.989	9.811	5.747
*P*	-	<0.001	<0.001	<0.001	<0.001

## DISCUSSION

In this study, infants in the control group were given routine nursing interventions, while those in the observation group received Kangaroo-mother care combined with infant touch. According to the results, there was no statistically significant difference in body height and weight between the two groups of infants at birth (*P*>0.05); and the body height and weight of the two groups of infants both increased at 3 days, 7 days and 30 days after birth, which were higher in the observation group than those in the control group (*P*<0.05). In addition, Kangaroo-mother care and infant touch can alleviate the infant’s anxiety, reduce energy consumption and stress response, comfort and calm the infant more significantly, thus promoting the infant’s growth and development.^10^,^11^ The two interventions can stabilize the physiological state of premature infants, alleviate their anxiety and stress reactions to comfort and calm them, thus stimulating their growth and development. In our study, the levels of zinc, iron, and prealbumin in the observation group were significantly higher than those in the control group at 30 days and 90 days after birth, respectively (*P*<0.05). Kangaroo-mother care combined with infant touch can increase the sense of security and intimacy of premature infants, and promote the secretion of neurotransmitters to improve the sleep quality and appetite of premature infants. Compared to routine nursing interventions, the combined intervention can better simulate the comfortable maternal environment for the fetus to enhance their security and intimacy, which is beneficial for the infant’s nutrient absorption and development.^12^

Furthermore, the good sleep quality rate of infants in the observation group was significantly higher than that in the control group (63.04% vs. 36.96%), while the poor sleep quality rate of infants in the former group was obviously lower than that in the latter group (8.70% vs. 26.09%), with statistically significant differences (*P*<0.05). Besides, the observation group had shorter night-time awakening duration and lower frequency of night-time awakenings than those in the control group, and longer night-time and day-time sleep duration than those in the latter group. It can be explained that Kangaroo-mother care combined with infant touch can enhance the security and intimacy for premature infants to warm, comfort and relax them, thereby realizing a deep sleep state. Meanwhile, the combined intervention can also promote infant-mother emotional communication to establish a parent-child relationship, which can strengthen their sense of security under the mother’s company, and thus improve the infant’s sleep quality.^13^,^14^ Compared to routine nursing interventions, Kangaroo-mother care combined with infant touch can stimulate the infant’s nervous system and gastrointestinal tract more effectively, thereby enhancing the digestion, absorption, and metabolism to comfort and relax them, thereby improving their sleep quality. The combined intervention can better simulate the comfortable maternal environment for the fetus to enhance their security and intimacy, resulting in an improved sleep quality. In addition, Kangaroo-mother care combined with infant touch can also promote infant-mother emotional communication, which can strengthen their sense of security under the mother’s company, and thus improve the infant’s sleep quality.

Premature infants refer to a group of infants born at a gestational age of <37 weeks^15^, among which infants with a gestational age of <34 weeks or a birth weight of <2,000g are defined as high-risk premature infants.^16^ Meanwhile, premature infants are also prone to neurodevelopmental disorders, learning difficulties, etc.^17^ The latest report revealed that there is about one million premature infants in China annually, with an increasing trend year by year, highlighting the significance of nursing interventions in the vital signs, health status, and psychological emotions of premature infants.^18^ However, it shall be acknowledged that there are still some shortcomings in the application of routine nursing interventions for premature infants. For example, this type of intervention focuses primarily on basic living needs of infants, such as feeding, cleaning, and warming, without additional attention paid to their psychological and emotional needs.^19^ Significantly, Kangaroo-mother care is a method of providing care for infants through skin-to-skin contact between the infant and the mother or father by stimulating the maternal environment for the fetus, thereby enhancing the infant’s sense of security and intimacy.^20^ Simultaneously, infant touch is a method of gently touching various parts of the infant and providing regular massage to the infant to promote the infant’s growth and development.^21^ It can promote the development of the infant’s nervous system, enhance immunity, stabilize emotions, promote sleep, etc.

### Limitations:

It includes small sample size, and the period from birth to discharge or three months of corrected age, is relatively short-term,Long-term follow-up data on the neurodevelopmental outcomes are lacking. Therefore, more samples may be included in studies, along with analyses using updated clinical indicators. In the future, we will conduct neurodevelopmental observation and research on premature infants, and do Long-term follow-up data on the neurodevelopmental outcomes, improvements shall be made to address these issues, thereby providing more data for clinical practices.

## CONCLUSIONS

Kangaroo-mother care combined with infant touch has a significant effect on premature infants, which can promote their physical development, improve their nutritional status and sleep quality.

### Authors’ Contributions:

**QD and** **HZ** carried out the studies, data collection, drafted the manuscript.

**MG:Z: S**tatistical analysis , participated in its design of study and critical review.

**HY and YM:** Acquisition, analysis, interpretation of data and drafted the manuscript.

**QD:** Literature search, critical analysis and is responsible for accuracy of the study.

All authors have read and approved the final manuscript.
